# Magnetoencephalography STOUT Method Adapted to Radiofrequency Thermocoagulation for MR-Negative Insular Epilepsy: A Case Report

**DOI:** 10.3389/fneur.2021.683299

**Published:** 2021-10-13

**Authors:** Kaiqiang Ma, Guoming Luan, Xiongfei Wang, Shen Luo, Lang Qin, Pengfei Teng, Yuguang Guan, Meng Zhao, Jing Wang, Mengyang Wang, Jia-Hong Gao

**Affiliations:** ^1^Department of Neurosurgery, Sanbo Brain Hospital, Capital Medical University, Beijing, China; ^2^Beijing Key Laboratory of Epilepsy, Epilepsy Center, Sanbo Brain Hospital, Capital Medical University, Beijing, China; ^3^Beijing Institute for Brain Disorders, Beijing, China; ^4^Center for MRI Research, Academy for Advanced Interdisciplinary Studies, Peking University, Beijing, China; ^5^Beijing City Key Laboratory for Medical Physics and Engineering, Institute of Heavy Ion Physics, School of Physics, Peking University, Beijing, China; ^6^Department of Neurology, Sanbo Brain Hospital, Capital Medical University, Beijing, China; ^7^McGovern Institute for Brain Research, Peking University, Beijing, China

**Keywords:** magnetoencephalography, STOUT, radiofrequency thermocoagulation, stereo-electroencephalography, insular epilepsy

## Abstract

Epilepsy is one of the most challenging neurologic diseases confronted by human society. Approximately 30–40% of the worldwide epilepsy patients are diagnosed with drug-resistant epilepsy and require pre-surgery evaluation. Magnetoencephalography (MEG) is a unique technology that provides optimal spatial-temporal resolution and has become a powerful non-invasive imaging modality that can localize the interictal spikes and guide the implantation of intracranial electrodes. Currently, the most widely used MEG source estimation method for clinical applications is equivalent current dipoles (ECD). However, ECD has difficulties in precisely locating deep sources such as insular lobe. In contrast to ECD, another MEG source estimation method named spatio-temporal unifying tomography (STOUT) with spatial sparsity has particular advantages in locating deep sources. In this case study, we recruited a 5 year-old female patient with insular lobe epilepsy and her seizure recurred in 1 year after receiving the radiofrequency thermocoagulation (RF-TC) therapy. The STOUT method was adopted to locate deep sources for identifying the epileptic foci in epilepsy evaluation. MEG STOUT method strongly supported a stereo-electroencephalographic (SEEG)-guided RF-TC operation, and the patient reported a satisfactory therapeutic effect. This case raises the possibility that STOUT method can be used particularly for the localization of deep sources, and successfully conducted RF-TC under the guidance of MEG STOUT results.

## Introduction

There has been an increasing interest in insular epilepsy since it was first described 70 years ago (1). The manifestation of insular seizure varies since the deeply located insular lobe is functionally connected to most brain areas (2). The diversity of clinical semiology of insular epilepsy, especially the magnetic resonance imaging (MRI)-negative insular epilepsy, often mischaracterizes the epileptogenic zone and leads to the failure of treatment (3).

More and more insular epilepsy patients are diagnosed as the stereo-electroencephalographic (SEEG) has been developed as a particularly effective tool for identifying seizure onset zone (4–6). Common therapies for insular epilepsy include insular cortex resections, bipolar electro-coagulation, and radiofrequency thermocoagulation (RF-TC). In contrast to insular cortex resections and bipolar electro-coagulation, RF-TC as a minimally invasion technique takes advantages of the already implanted SEEG electrodes and has quite few complications (7). However, due to the limited scope of RF-TC (8), the success rate of RF-TC for insular epilepsy has never been close to that of resection surgery and it is just regarded as a palliative treatment. As the success of RF-TC for hypothalamic hamartomas (HH), periventricular nodular heterotopias (PNH), and small focal cortical dysplasias (FCD) depends on high-resolution MRI, it should expect that the success rate of RF-TC for insular epilepsy will be increased when ablations are performed based on the scope of a given epileptogenic lesion.

In this paper, a case of recurrent insular epilepsy after RF-TC is reported. As a unique technology, magnetoencephalography (MEG) can provide optimal spatial-temporal resolution and localize the interictal spikes and guide the implantation of intracranial electrodes. Given the equivalent current dipoles (ECD), the most widely used MEG source estimation method, often fail to locate deep sources, we adopted spatio-temporal unifying tomography (STOUT) to delineate the scope of epileptogenic lesions within insular region. Guided by the results generated with STOUT, the patient received RF-TC operation and has now already achieved seizure free.

## Case Presentation

A 5 year-old right-handed girl with no family history was re-admitted to our epilepsy center because the seizures recurred 1 year after first ablation. When she was 3 years old, the patient was admitted to our hospital for first evaluation as a 5-month history of seizures. The form of seizures was a loss of consciousness followed by her head and eyes turned to the right and then tonic–clonic seizure of right limbs. Seizures were not controlled after taking at least three anti-seizure medications (oxcarbazepine, sodium valproate, and lacosamide), and the frequency of seizures gradually increased to about 7–10 times per day.

**Figure 1:**
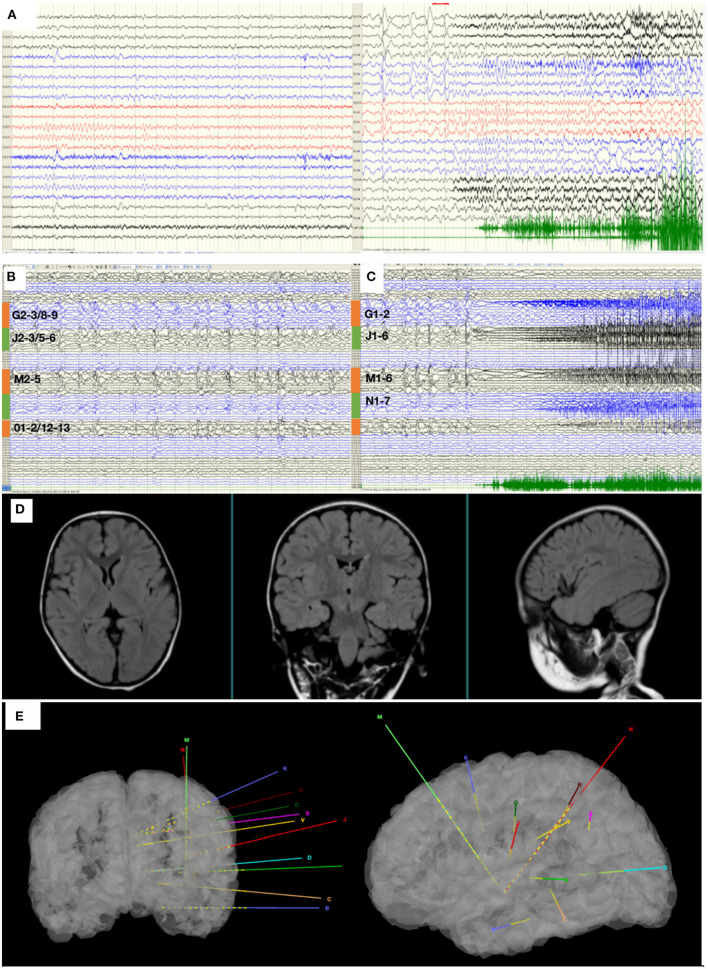
**(A)** The interictal and ictal scalp VEEG. Scalp VEEG showed seizure onset with stiffening of right limb, apnea, salivation, waving of left limb. **(B)** SEEG showed all the inter-ictal discharges were located in insular-opercular cortex (J2-3/5-6, M2-5, G2-3/8-9, 01-2/12-13). **(C)** SEEG showed that the seizures onset zones originated from the insular-opercular cortex (J1-6, G1-2, M1-6, N1-7) with spike-wave in fast activities. The Semiology is stiffening of right limb, apnea, salivation, waving of left limb. **(D)** MRI images of the patient before the first intracranial electrode implantation. **(E)** The position of twelve electrodes.

Epilepsy evaluation included MRI, video-electroencephalographic (VEEG) recordings, MEG recording (with ECD source estimation), and 18F-fluorodeoxyglucose positron emission tomography (18F-FDG–PET) scan. The VEEG recorded several seizures without heart rate abnormalities, and results of VEEG are displayed in Figure 1A. The MRI showed no obvious abnormalities (Figure 1D), which was evaluated by a neuroradiologist and neurosurgeon. Large hypometabolic regions surrounding left sylvian fissure can be seen on PET. After evaluation, a case of focal epilepsy surrounding left sylvian fissure was considered. To validate the seizure onset zone, 12 intracranial electrodes surrounding left sylvian fissure were stereotaxically implanted and their positions are shown in Figure 1E. SEEG electrodes (5–18 contacts, diameter: 0.8 mm; length: 2 mm; 1.5 mm apart) were manufactured by Alcis, Besancon, France. The SEEG monitoring continued for 2 days during which 13 seizures were recorded, and the results of SEEG are displayed in Figures 1B,C. The epileptogenic zone was located in the third insular short gyrus, the insular long gyrus, and the parietal opercula.

According to SEEG results, RF-TC therapy targeting the ictal site was designed (J1-6, G1-2, M1-6, N1-7). RF-TC with an output power (3.5 W) and ablation time (120 s) resulted in successful thermo-conduction. RF-TC generator system was R-2000b (Beiqi, Beijing, China).

**Figure 2:**
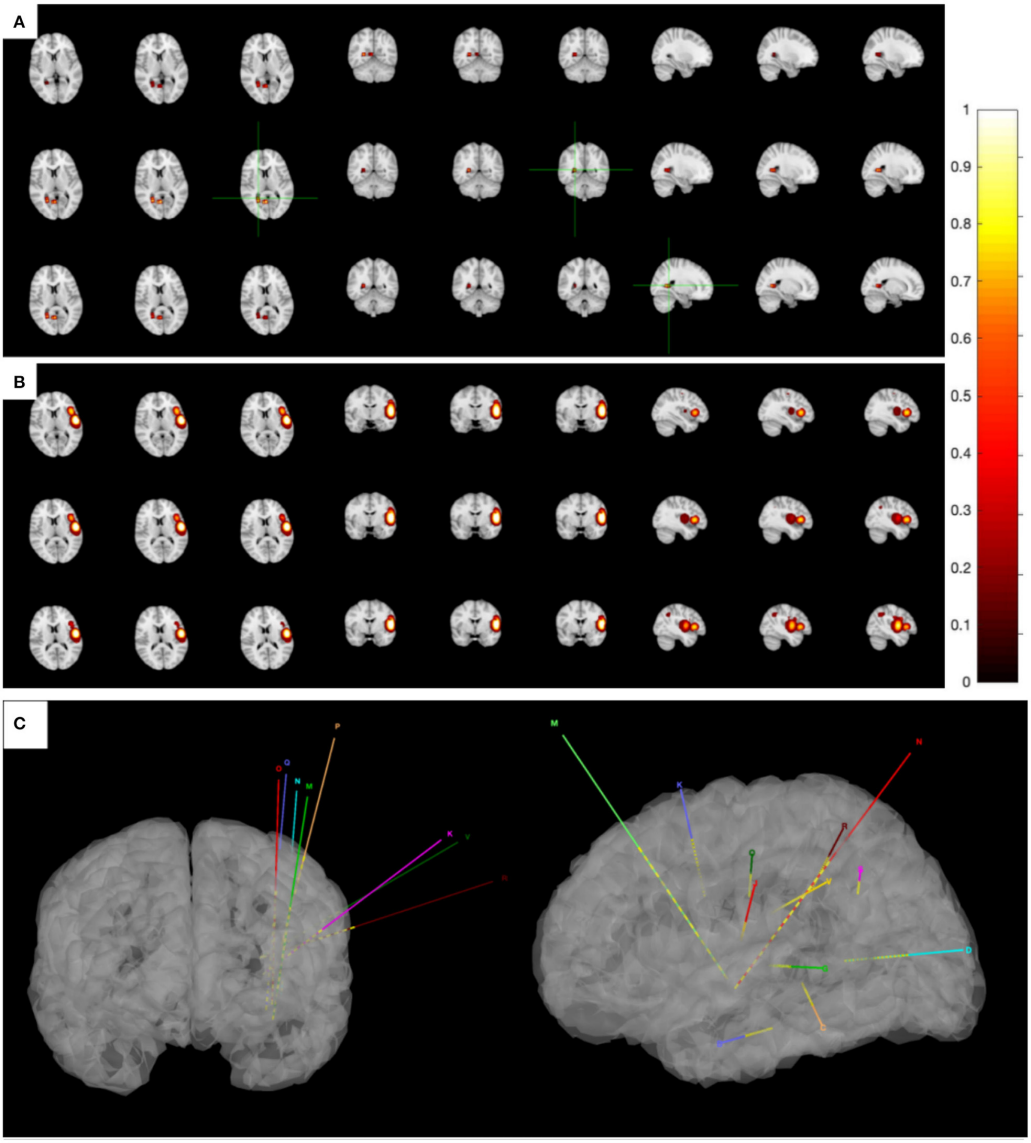
**(A)** The MEG ECD method delineated epileptic zone at right posterior cingulate cortex. **(B)** The MEG STOUT method delineated epileptic zone at the left insular long gyrus of island and the parietal opercula. Thirteen spikes were marked in this patient and Source localization was conducted using ECD and STOUT on each spike within a −100 to 100 ms time window around the peak spike signal. All results displayed over the corticalsur face are thresholded at 50% of the maximum amplitude. As ECD method assumes that a small number of focal sources exist that can be equivalent to a few current dipoles in the brain, the result of MEG ECD method is localized to right posterior cingulate cortex. And the STOUT method is localized to the left insular long gyrus of island and the parietal opercula through localization bias compensation. **(C)** The position of eight intracranial electrodes.

**Figure 3:**
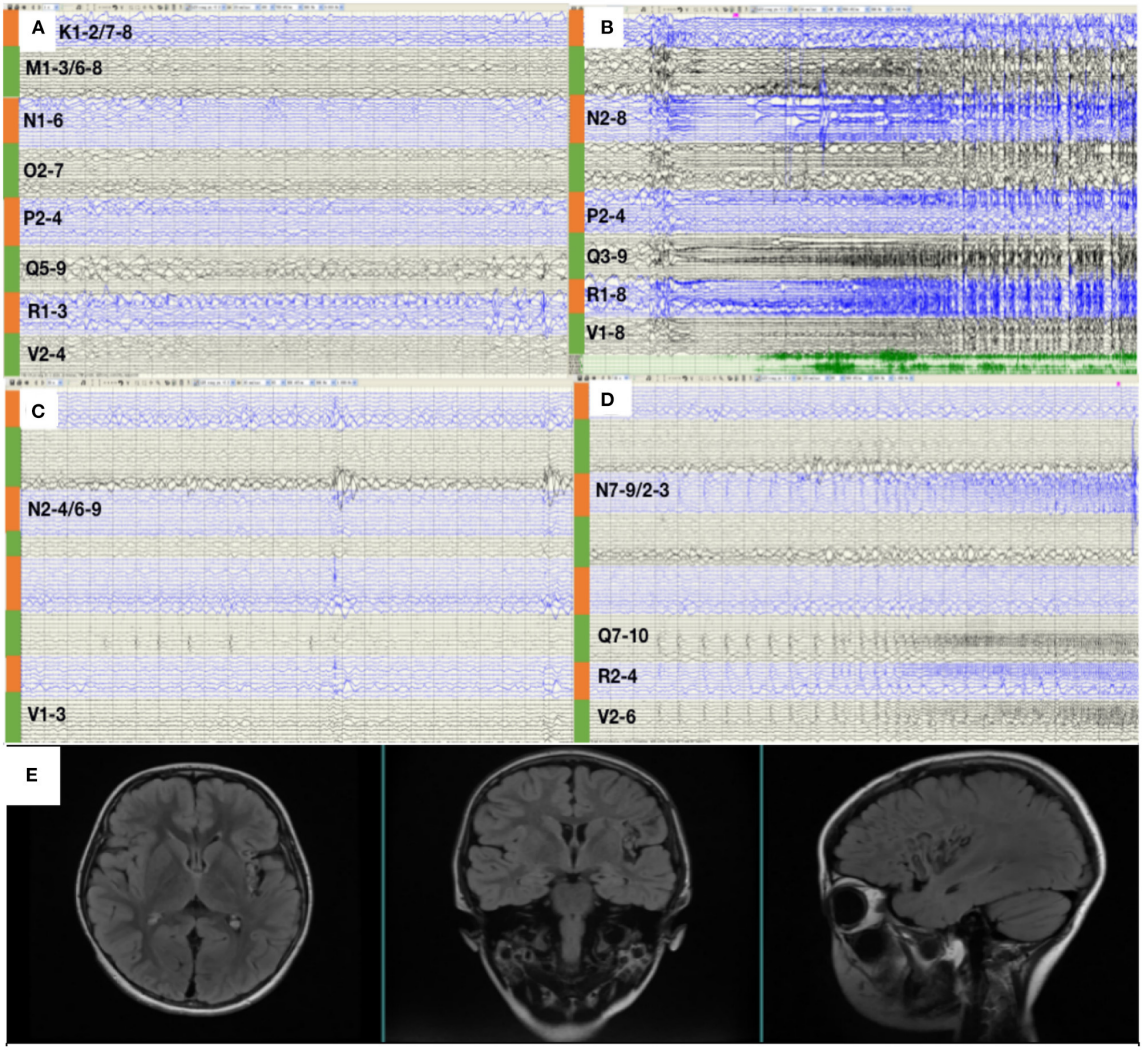
**(A)** SEEG showed that all the inter-ictal discharges were located in the insular and insular-opercular cortex (N1-6, 02-7, P2-4, R1-3, V2-4, Q5-9, K1-2/7-8, M1-3/6-8). **(B)** The onset zone also originated from the insular and insular-opercular cortex (Q3-9, R1-8, N2-8, V2-4, P2-4) with spike—waves in fast activities. **(C)** SEEGs for the inter-ictal discharges were located in the insula (N2-4/6-9,V1-3). **(D)** The onset zone originated from the insular and insular-opercular cortex (N7-9/2-3, Q7-10, V2-6, R2-4) with spike—waves in fast activities. **(E)** Images 1 year after RF-TC showed a satisfying ablation.

While seizures ceased in 1 month after ablation, the patient regained seizures in about a year, and the antiepileptic medication was taken at the original dose. This time, the patient was again admitted to our hospital for further evaluation. The VEEG monitor recorded for 24 h, and six seizures occurred. The MRI showed no obvious abnormalities. The hypometabolic regions surrounding left sylvian fissure can be seen on PET and is more localized than the previous one. MEG ECD showed that the epileptogenic zone was located in the right posterior cingulate cortex (Figure 2A). Taken together these results, we diagnosed that the epileptogenic zone should be located in insula and insulo-opercular areas. Eight intracranial electrodes densely covering the insula and insulo-opercular were implanted, and their positions are shown in Figure 2C. Wearing SEEGs, the patient was monitored for 2 days, and 10 seizures were captured. The semiology was stiffening of right limb followed by left limb complex movement, apnea, and laryngeal myoclonus. Figure 3A shows that SEEGs for all the inter-ictal discharges were located in the insular and insulo-opercular cortex (N1-6, O2-7, P2-4, R1-3, V2-4, Q5-9, K1-2/7-8, M1-3/6-8). The onset zone also originated from the insular and insulo-opercular cortex (Q3-9, R1-8, N2-8, V2-4, P2-4) with spike–waves in fast activities (Figure 3B).

RF-TC therapy (Q2-10, R1-9, N1-9, V1-5, P1-5) targeting onset zone was designed and conducted with an output power (3.5 W) and ablation time (120 s), after which the patients were monitored wearing SEEGs for 2 days and four seizures were captured. The semiology of seizures changed to an aura of abdominal discomfort, and then decreased body activity, apnea, and tonic clonus of the proximal right limb. Figure 3C shows that SEEGs for the inter-ictal discharges were located in the insula (N2-4/6-9, V1-3). The onset zone originated from the insular and insulo-opercular cortex (N7-9/2-3, Q7-10, V2-6, R2-4) with spike-waves in fast activities (Figure 3D).

Since the scope of ablation guided by SEEG was obviously insufficient, new guiding strategies for RF-TC shall be developed. As a result, MEG STOUT was adopted to further clarify the location of epileptogenic zone. The resting-state MEG date was record (0.1–300 z pass filter, 1-kHz sample rate) for 90 min after sleep deprivation using 306-channel VectorviewTM MEG system. After recording, the date was processed as follows: First, denoising operation to reduce environmental noise. Second, registering the MEG date with the MRI coordinate systems and marking interictal spikes. The spike selection criteria has been described extensively by Mohamed et al. (9). Briefly, spikes were marked on MEG by an expert epileptologist, who then selected those showing a clear dipolar magnetic field; this criterion ensures selected spikes have focal generators. Thirteen spikes were marked in this patient. Third, the STOUT solution was performed for datesets. Source localization was conducted using ECD and STOUT on each spike within a −100 to 100 ms time window around the peak spike signal, when the noise covariance estimation selected a 2,000 ms time window as baseline. The MEG STOUT method delineates the epileptic zone in the insular long gyrus, and the parietal opercula (Figure 2B) covered the epileptogenic zone determined by the original SEEG. We compared the STOUT source localization with the epileptogenic zone identified by SEEG and found the STOUT source localization can completely cover the lesions of ablation. Also, the epileptogenic zone mainly located in the insular long gyrus of island and the parietal opercula. The insufficient ablation of insular long gyrus and the parietal opercula seizures led to the dissatisfied therapeutic effect. According to the STOUT source localization, RF-TC (V5-7, V4-N8, V4-N9, V5-N8, V5-N9, V3-Q8, V3-Q9, V4-Q8, V4-Q9, R2-Q6, R3-Q6, R4-Q6, R2-Q7, R3-Q7, R3-Q8) treatment was performed again. The RF-TC was performed between two adjacent contacts of the different electrodes to achieve full ablation, after which a 24 h SEEG records showed no inter-ictal discharges. Of note, seizures ceased right after the ablation. Post-ablation complications for this patient included clumsy speech and limp, but these complications recovered within half a month. No intelligence decline was observed, and the parent was satisfied with the treatment. After the ablation, three anti-seizure medications (oxcarbazepine, sodium valproate, and lacosamide) were given as the postoperative antiepileptic drug. At the 1 year follow-up, there was no sign of relapse and VEEG showed no inter-ictal discharges. MRI image of the patient showed a satisfying ablation and was shown in Figure 3E. The timeline with relevant data from the episode of care is shown in Table 1.

**Table 1:** Patient's clinical data of each attack or therapy.

**Attack or therapy**	**1**	**2**	**3**	**4**	**5**
Time from disease onset	onset	+5 months	+18months	+24months	+24months
Age (years)	3	3.5	4.5	5	5
Semiology	Loss of consciousness, head and eyes turned to the right and then tonic-clonic seizure of right limbs	Stiffening of right limb, apnea, salivation, waving of left limb	Stiffening of right limb, left limb complex movement, apnea, laryngeal myoclonus	Stiffening of right limb, left limb complex movement, apnea, laryngeal myoclonus	Abdominal discomfort, decreased body activity, apnea, tonic clonus of the proximal right limb
Medicine	Oxcarbazepine	Oxcarbazepine, sodium valproate, Lacosamide	Oxcarbazepine, Sodium valproate, Lacosamide	Oxcarbazepine, sodium valproate, Lacosamide	Oxcarbazepine, sodium valproate, Lacosamide
Therapy	Medicine	Medicine+RF-TC	Medicine	Medicine+RF-TC	Medicine+RF-TC guided by STOUT
Clinical response to therapy	Relief	Seizures ceased in 1 month after ablation	Recur	Relief	Seizure free

## Discussion

SEEG was proposed by Talairach et al. (10). As a minimal invasive method, it offers a unique means of accurately mapping the epileptogenic network in pre-surgical evaluations of epilepsy. Moreover, SEEG-guided RF-TC, which ablates epileptogenic zone directly through the recording electrodes according to SEEGs evidence, is considered as a minimally invasive treatment with notable preservation of neurocognitive functions. SEEG-guided RF-TC is considered as a palliative approach, and its principle often referred to selectively destroying epileptogenic zone or the critical nodes of epileptogenic networks (7). MR-positive epileptic foci including HH, PNH, and FCD type II (11) demonstrated satisfactory results after ablation. For MR-negative lesions, especially MR-negative insular lesions, the ablation effect is not satisfactory. It has been reported that only 11% of the patients were persistently seizure-free and 41% were responders (12). So STOUT method was used to clarify the scope of the epileptogenic zone.

As a non-invasive measure, MEG has high value for epilepsy patients' pre-surgical evaluation (13), with the ECD method being a standard method for MEG to locate interictal epileptiform discharges (14). Moreover, MEG can assist intracranial electrode placement planning (15–17). The ability of ECD method to delineate the epileptic zone is very limited due to (1) difficulties in localizing extended sources and (2) an error in the sensitivity of the dipole to the deep location (18). In this insular epilepsy case, the deep source location with MEG ECD was inaccurate.

STOUT method as a minimum L1-norm solution has spatial sparsity (19), which is different from the minimum L2 norm estimation, a distributed source imaging technology that leads to the low spatial resolution of the reconstructed image and the overestimated area of the active region boundary. STOUT approach combines the main advantages of Sparse Basis Field Expansions (S-FLEX) (20) and Time–Frequency Mixed-Norm Estimates (TF MxNE) (21). S-FLEX expresses current density as a linear combination (20), which is locally smooth but space-constrained spatial basis function. TF MxNE decomposes the current density into the time basis function and the corresponding coefficient. Similarly, as a combination of S-FLEX and TF-MxNE, STOUT expresses the current density as a linear combination of spatio-temporal basis functions.

To compensate for localization bias, STOUT adopts a diagonal weight matrix


$$\Psi (\text{i},\text{i})\text{=}\sqrt{{\left({\text{||L}}_{\text{x}}(\cdot ,{\text{i||}}_{\text{2}}^{\text{2}}\;+\;{\text{||L}}_{\text{y}}(\cdot ,{\text{i||}}_{\text{2}}^{\text{2}}\text{ + }{\text{||L}}_{\text{z}}(\cdot ,{\text{i||}}_{\text{2}}^{\text{2}}\;\right)}^{\zeta }}.$$

Parameters determine the intensity of depth compensation. When ζ = 0, there is no depth bias compensation, while ζ = 1 leads to full compensation. Therefore, the accuracy of deep source localization is greatly improved. For reference, MEG STOUT method was used to locate the deep source in this case, assisting in delineating the epileptogenic zone. Compared with the other two source estimation methods, the accuracy of the STOUT method in clinical used was described by Zheng et al. (22).

In this article, we used MEG STOUT to locate the deep source of the insula. Compared with the first ablation location, post-surgery outcomes verified that the STOUT location was accurate, and the RF-TC guided by MEG STOUT achieved satisfactory treatment results. Still, the accuracy of MEG STOUT method needs to be verified by large samples in clinical applications. Conclusively, the importance of this case is that we gave MEG STOUT method as a potentially optimal solution to locate deep sources, which can effectively assist RF-TC by accurately locating the epileptogenic zone. By continuously improving the accuracy of MEG source positioning methods, in the future, it may be possible to achieve precise positioning of epileptogenic zone through non-invasive methods.

## Data Availability Statement

The original contributions presented in the study are included in the article/supplementary material, further inquiries can be directed to the corresponding author/s.

## Ethics Statement

The studies involving human participants were reviewed and approved by the Ethics Committee of Sanbo Brain Hospital, Capital Medical University. Written informed consent to participate in this study was provided by the participants' legal guardian/next of kin. Written informed consent was obtained from the minor(s)' legal guardian/next of kin for the publication of any potentially identifiable images or data included in this article.

## Author Contributions

KM collected the patient information, conceptualized and designed the study, performed the SEEG-guided RF-TC, and drafted the manuscript. SL, LQ, XW, and PT conducted the computational calculations. MW and JW performed the clinical data analyses. GL, YG, and MZ conducted the electrode implantation. MW and JG critically revised the manuscript. All authors contributed to the article and approved the submitted version.

## Funding

This work was supported by the National Natural Science Foundation of China (81790650, 81790654, and 81790651).

## Conflict of Interest

The authors declare that the research was conducted in the absence of any commercial or financial relationships that could be construed as a potential conflict of interest.

## Publisher's Note

All claims expressed in this article are solely those of the authors and do not necessarily represent those of their affiliated organizations, or those of the publisher, the editors and the reviewers. Any product that may be evaluated in this article, or claim that may be made by its manufacturer, is not guaranteed or endorsed by the publisher.
